# Grazing effects on ecosystem CO_2_ fluxes differ among temperate steppe types in Eurasia

**DOI:** 10.1038/srep29028

**Published:** 2016-07-01

**Authors:** Longyu Hou, Yan Liu, Jiancai Du, Mingya Wang, Hui Wang, Peisheng Mao

**Affiliations:** 1 Forage Seed Lab, China Agricultural University, Beijing Key Laboratory of Grassland Science, Beijing 100193, China; 2 State Key Laboratory of Tree Genetics and Breeding, Research Institute of Forestry, Chinese Academy of Forestry, Beijing 100091, China; 3 Institute of Grassland Research, Chinese Academy of Agricultural Sciences, Hohhot 010010, China

## Abstract

Grassland ecosystems play a critical role in regulating CO_2_ fluxes into and out of the Earth’s surface. Whereas previous studies have often addressed single fluxes of CO_2_ separately, few have addressed the relation among and controls of multiple CO_2_ sub-fluxes simultaneously. In this study, we examined the relation among and controls of individual CO_2_ fluxes (i.e., GEP, NEP, SR, ER, CR) in three contrasting temperate steppes of north China, as affected by livestock grazing. Our findings show that climatic controls of the seasonal patterns in CO_2_ fluxes were both individual flux- and steppe type-specific, with significant grazing impacts observed for canopy respiration only. In contrast, climatic controls of the annual patterns were only individual flux-specific, with minor grazing impacts on the individual fluxes. Grazing significantly reduced the mean annual soil respiration rate in the typical and desert steppes, but significantly enhanced both soil and canopy respiration in the meadow steppe. Our study suggests that a reassessment of the role of livestock grazing in regulating GHG exchanges is imperative in future studies.

CO_2_ is the single most important greenhouse gas in the atmosphere, contributing approximately 56% of the total global warming strength^1^. Accurately distinguishing constituent CO_2_ fluxes and their responses to human activity remains a challenge to ecologists^2^. Grassland soil represents one of the major carbon reserves and plays a critical role in regulating the emission and uptake of CO_2_ into and out of the Earth’s surface^3^. Although generally consistent in their production and emission processes and mechanisms in grassland soils, the magnitudes and patterns of CO_2_ exchanges are quite type- and site-specific^4^. In recent decades, a large number of studies have been carried out to examine the relation among and controls of CO_2_ fluxes in grassland ecosystems, as affected by human activities^5^,^6^,^7^. However, these experiments often examined the fluxes and controls of CO_2_–related processes separately, while few have assessed the relation among and balance of multiple sub-CO_2_ fluxes as a whole.

As arguably the most important human influence on grasslands, livestock grazing has been evidenced as an important regulatory factor in CO_2_ exchanges^8^,^9^,^10^. The biophysical and chemical controls and responses of CO_2_ sub-fluxes to grazing may also vary across steppe types due to site-specific grazing histories^11^. However, the patterns of and relevant mechanisms underlying the relation among and controls of multi-CO_2_ fluxes in various grassland types, as affected by land use pattern (grazing vs no grazing), are largely unknown.

Grasslands account for 41.7% of China’s total land area and are mainly distributed in arid and semi-arid regions. The steppes of Inner Mongolia (87 million hectares), which include three major types (i.e., meadow, typical, and desert steppes along a decreasing rainfall gradient), are an important component of China’s grasslands^12^. In this study, consecutive three-year field observations of CO_2_ exchanges in three contrasting temperate steppes in north China were conducted to examine the seasonal and annual relation among and biophysical controls of individual CO_2_ fluxes in response to livestock grazing, at a regional scale.

## Results

### Seasonal patterns and controls

**Figure 1:**
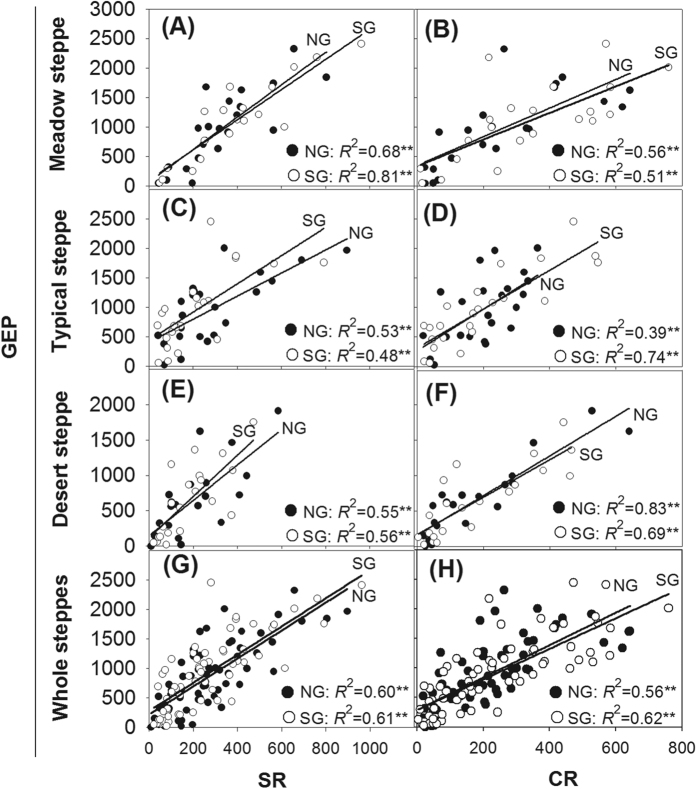
Relations in the seasonal rate of gross ecosystem photosynthesis (GEP) and soil respiration (SR) (**A,C,E,G**) and between the seasonal rates of GEP and canopy respiration (CR) (**B,D,F,H**) for the meadow, typical, desert, and whole steppes, under no grazing (NG) and summer grazing (SG). **Indicates significance at the *P* < 0.01 level.

Across the growing seasons, significant positive relations were found between gross ecosystem photosynthesis and air temperature, but only in the grazed meadow steppe and the ungrazed typical steppe (Table 1, both P < 0.05). No significant seasonal relations between canopy respiration and single or combined climatic factors were detected for any steppe type (Table 1, all P > 0.05). However, grazing significantly enhanced the seasonal dependence of canopy respiration on air temperature in the meadow steppe (Table 1, P < 0.01), whereas it significantly enhanced the seasonal dependence of canopy respiration on soil moisture in the typical and desert steppes (Table 1, P < 0.01, P < 0.05, respectively). Soil moisture and temperature were the major single factors controlling the seasonal dynamics of soil respiration at the desert and typical sites, respectively, whereas the combined effect of soil moisture and temperature played a major role in the meadow steppe. Summer grazing reduced the seasonal dependence of soil respiration on soil moisture to some extent in the meadow steppe, but this was increased in the typical and desert steppes (Table 1). In addition, significant positive relations were found between gross ecosystem photosynthesis and either soil respiration or canopy respiration within each steppe or in all three sites taken together, regardless of grazing (Fig. 1, all P < 0.01).

### Annual patterns and controls

Based on annual rates, gross ecosystem photosynthesis was significantly negatively related to air temperature (Table 2, P < 0.01), whereas soil respiration and canopy respiration were significantly positively related to soil moisture (Table 2, P < 0.01, P < 0.05, respectively). Grazing greatly reduced the annual dependence of gross ecosystem photosynthesis on air temperature in the meadow steppe, whereas it substantially enhanced the dependence of soil and canopy respiration on soil moisture in the typical and desert steppes (Table 2). Significant positive relations were found between soil or canopy respiration and peak canopy biomass, irrespective of grazing (Table 2, all P < 0.05).

### Grazing effects on bulk fluxes

**Figure 2:**
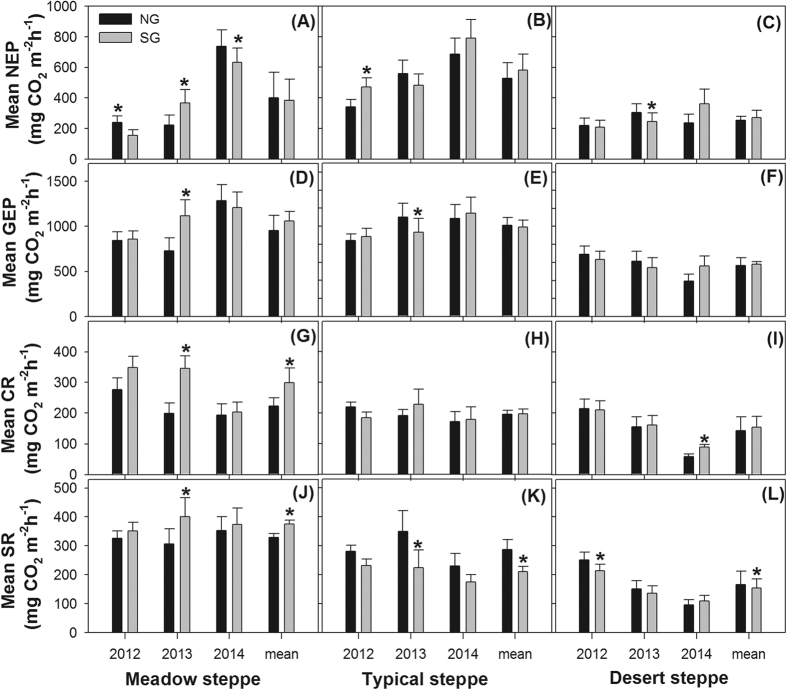
Annual mean rates of net ecosystem photosynthesis (NEP), gross ecosystem photosynthesis (GEP), canopy respiration (CR), and soil respiration (SR) under the no grazing (NG) and summer grazing (SG) treatments in the meadow (**A,D,G,J**), typical (**B,E,H,K**) and desert steppes (**C,F,I,L**). The data are represented as the mean ± standard error. *Indicates a significant difference (*P* < 0.05) between summer grazing and no grazing, according to the paired-sample t-test.

The typical steppe and the meadow steppe showed comparably high mean annual gross ecosystem photosynthesis rates, whereas the meadow steppe displayed the highest mean annual ecosystem respiration (SR plus CR), resulting in a carbon sink sequence of typical > meadow > desert steppes (Fig. 2). Grazing significantly reduced the mean annual soil respiration rates in the typical and desert steppes (Fig. 2, both P < 0.05), but significantly enhanced the mean annual soil respiration and canopy respiration in the meadow steppe (Fig. 2, both P < 0.05). However, no significant grazing effects on either net ecosystem photosynthesis nor gross ecosystem photosynthesis were found in our study (Fig. 2, all P > 0.05).

Taken together, our results from the multivariate analyses indicate that steppe type was the major contributor to the variances in the annual rates of all the carbon fluxes, whereas year had less significant effects for only the net ecosystem photosynthesis and ecosystem respiration, with summer grazing having no significant effects in this respect (Table 3).

## Discussion

No factors were significantly related to NEP, a result that agrees with previous studies^13^,^14^. Klumpp *et al*. indicated that the factors predominantly affecting NEP were plant and soil community structure and/or soil organic matter (SOM) compartments^13^. Risch and Frank indicated that the plant community structure was the best predictor of NEP^14^. Cao *et al*. indicated that although grazing did not change the SOM content, it changed its compartments^15^. Thus, not only biomass and SOM content but also plant community structure and soil organic compartments can be considered driving factors affecting NEP in future studies. A lack in seasonal relations between gross ecosystem photosynthesis and any climatic factors in this study are consistent with several previous studies^16^. A possible explanation is that nutrient availability was the major limiting factor regulating the gross ecosystem photosynthesis rate, in addition to light radiation, at the study area^17^. In other studies, the composition of plant species also had effects on GEP^18^, and grazing significantly changed plant composition in each steppe type^16^,^19^,^20^. Grazing induced higher CO_2_ exchange rates in the early stages of the growing season for the spring green-up, but decreased the CO_2_ exchange rate in the middle and late stages of the growing season; these results confused the effects of climatic factors on GEP^19^. The grazing-enhanced seasonal dependence of canopy respiration on air temperature and/or soil moisture might be related to livestock herbivory of photosynthesis-effective leaves, which leaves behind large amounts of stems and basal tillers that mainly carry out maintenance respiration, which is more dependent on climatic conditions^18^. In addition, the re-growth ability of plants was higher in summer grazing plots than that in no grazing plots as a result of accelerated nutrient cycling^21^. Our findings that climatic factors controlling the seasonal soil respiration patterns differ greatly among steppe types were largely related to the differences in the soil moisture regimes among these steppes^4^. Soil respiration is usually more soil moisture-dependent at drier sites (i.e., the desert steppe), whereas root respiration accounts for a considerable portion of the total soil respiration at wetter sites (i.e., the typical and meadow steppes) and is highly dependent on soil temperature^7^, leading to significant relations between soil respiration and soil moisture at the desert steppe and between soil respiration and soil temperature at the typical and meadow steppes (Table 1). Alterations in seasonal climate-soil respiration relations in response to grazing can be attributable to the effects of grazing on canopy cover and ground litter accumulation, which could have imposed effects on the seasonal patterns in soil moisture and/or temperature to a certain extent. Significant positive relations between gross ecosystem photosynthesis and soil respiration in this study (Fig. 1) were mainly due to the direct dependence of soil respiration on newly photosynthesized carbohydrates^5^,^22^,^23^,^24^.

The negative relation between the annual gross ecosystem photosynthesis rate and annual air temperature (Table 2) is a result of several associated processes. First, higher air temperature was associated with lower annual rainfall in the growing season in the study area^4^, leading to photoinhibition due to water and nutrient deficiencies^25^. For example, lower annual gross ecosystem photosynthesis rate resulted from higher air temperature and lower soil moisture in desert steppe, while opposite result was showed in meadow steppe (Fig. 2, Table 4). Second, the optimal temperature for plant growth is relatively low (approximately 20 °C) in C_3_ species-dominant temperate steppes^26^, above which photoinhibition also occurs due to accumulation of photosynthates, resulting in up to a 30% loss of net photosynthesis via enhanced photorespiration^25^. Annually-based positive relations of both soil and canopy respiration to soil moisture were largely due to the positive dependence of net primary productivity and soil organic matter decomposition on soil moisture in the area^4^, especially given that annual and between-site variation in temperature were rather small compared to that of soil moisture. Similar results have been reported in grasslands^4^, shrubland sites^27^ and forests^28^. The grazing-reduced dependence of gross ecosystem photosynthesis on air temperature at the meadow steppe and the grazing-enhanced dependence of soil and canopy respiration on soil moisture were mainly related to alterations in soil biophysical regimes due to grazing herbivory and trampling, improvement of light supply to the basal leaves to a certain extent, and partially also to stimulate plant growth^18^ and requirement-induced transportation of water and nutrients to the canopy from the soil in response to grazing^25^.

The highest mean annual gross ecosystem photosynthesis rate in the meadow steppe was mainly associated with the highest leaf area index (LAI) and leaf area duration (LAD) due to more favorable soil moisture and nutrient regimes (Table 4), as was the highest mean annual canopy respiration^25^. The highest soil respiration occurring in the meadow steppe was mainly related to the highest mean annual soil moisture, total soil carbon and nitrogen contents, and C/N ratio^29^ (Table 4). The significantly reduced mean annual soil respiration as a result of grazing in the typical and desert steppe is highly consistent with most previous studies^30^,^31^,^32^, and was related to significant decreases in litter accumulation and root biomass due to grazing at these sites^7^,^29^. In contrast, the mechanism for enhanced soil respiration as a result of grazing in the meadow steppe involves increased root biomass and N availability and a lowered to neutral pH value (Table 4). Indeed, a few previous studies have reported increased soil CO_2_ efflux in response to grazing in a typical steppe of north China^33^, a semi-arid mixed-grass prairie^11^, a tall-grass prairie with high rainfall^34^, and a shortgrass steppe in Colorado^35^. A lack of grazing effects on gross ecosystem photosynthesis in our study (Fig. 2) presumably occurred because decreases in LAI due to herbivory had been compensated by the improvement in the light regime for basal leaves^25^ via increased LAD, or by grazing-stimulated transportation of water and nutrients for plant growth^18^. Odriozola *et al*.^21^ indicated that grazing accelerates nutrient cycling and changes forage quality.

Our findings suggest that grazing can decrease soil respiration in arid and semi-arid steppes to some extent (Fig. 2), which confirmed the latest report by Han *et al*.^16^, and is conducive to mitigating the magnitude of changes in CO_2_ emission and global change effects in arid lands worldwide. In contrast, grazing can increase soil respiration in meadow steppe by increasing the root biomass, soil organic matter, and total nitrogen content^19^ (Table 4). The effect of SG on NEP varied among years, such as decreasing NEP in 2012, and increasing NEP in 2013 and 2014. In their latest published study, Shao *et al*.^36^ indicated that grazing can induce opposite effects on NEP in two consecutive years in desert soil through eddy-covariance systems and that the driving factors for these effects also changed. Heavy grazing decreased as the management of grassland use strengthened, especially in China. This can affect CO_2_ fluxes to some extent. Furthermore, in our experiment and in Shao *et al*.^36^, the effect of grazing on NEP changed in different years for the same sites. Questions remain regarding whether the interannual variation in NEP induced by grazing should be considered in estimating global CO_2_ exchange and how these results should be used in estimating the grazing effects on CO_2_ fluxes in grassland ecosystems. More field experiments are required to address these questions. In conclusion, based on our experiment and other recent studies^36^, a reassessment of the role of livestock grazing in regulating greenhouse gas exchanges is imperative in future studies.

## Methods

### Study sites

The study area is characterized by a temperate continental monsoon climate. The meadow steppe site (120.3 °N, 45.1 °E) was located in the middle northeast of the Inner Mongolia Plateau, with an elevation of 656 m ASL. The 30-year (1971–2000) mean annual temperature and precipitation were 2.1 °C and 395 mm, respectively, with a frost-free period of 106 days. The soil is mainly Typical Kastanozem (The Food and Agriculture Organization of the United Nations (FAO) soil classification). Vegetation is dominated by *Leymus chinensis, Filifolium sibirricum,* and *Carex* spp. The typical steppe site (116.7 °N, 43.6 °E; 1268 m ASL) lies in the central part of the Inner Mongolia Plateau. The average annual temperature is −0.3 °C, with a frost-free period of 98 days. The mean annual precipitation is 293 mm (1971–2000). Vegetation is dominated by *Stipa grandis, Agropyron michnoi, Cleistogenes squarossa*, and other bunchgrasses. The soil is classified as a calcic Chernozem. The desert steppe site (111.9 N, 41.8 E; 1428 m ASL) is located in the mid-southwest of the Inner Mongolia Plateau, being characterized by a short growing season and long, cold winters, with a frost-free period of 75 days and a mean annual temperature of 3.1 °C. The average annual precipitation is approximately 175 mm (1971–2000). Vegetation is dominated by *Stipa breviflora, Artemisia frigida, Thymus serpyllum*, and *Caragana* shrubs. The dominant soil type is a light-colored Chernozemic soil (sandy chestnut soil with a loamy sand texture).

### Experimental design

Randomly selected paired plots of summer grazed (SG) and no grazing (NG) areas of 100 × 100 m^2^ were established at each of the three sites. Summer grazed plots were strictly restricted in terms of stocking rate and time period (0.5 sheep units per hm^2^ during the growing season). The no-grazing areas were enclosed to prohibit grazing. The effects of grazing on carbon dioxide exchanges [i.e., net ecosystem photosynthesis (NEP), ecosystem respiration (ER), and soil respiration (SR)] and their driving factors were investigated. Ten days prior to taking formal measurements, nine bases (length × width × height = 0.5 m × 0.5 m × 0.1 m) were randomly installed at each site in each plot (SG vs NG) 10 cm into the soil, of which three bases were fixed for the measurement of net ecosystem photosynthesis, ecosystem respiration, and soil respiration. Air and soil temperatures, soil moisture, and maximum shoot biomass were also measured on each sampling occasion.

### Measurements of CO_2_ fluxes

CO_2_ fluxes were measured from May 2012 through September 2014 using the static chamber method. Gas samples were collected every ten days during May through September in 2012 and monthly at other times. Net ecosystem photosynthesis (NEP) was measured using transparent chambers (length × width × height = 0.5 m × 0.5 m × 0.4 m), and the ecosystem respiration (ER) and soil respiration (SR) were measured using opaque chambers (length × width × height = 0.5 m × 0.5 m × 0.25 m). Detailed descriptions of transparent and opaque chambers were presented by Zhang *et al*.^37^ and Hou *et al*.^38^, respectively. Canopy biomass within the base frame was clipped at the ground level several days before gas sampling to measure soil respiration. Chamber headspace gas samples were collected at 0, 10, 20, and 30 min time intervals for the opaque chamber (measuring ER and SR) and at 0, 1, 2, 3 min time intervals for the transparent chamber (measuring NEP) using 100-ml polypropylene syringes in the morning (9:00–10:00). The gases were then injected into 100 ml sealed airbags. The airbags were transported to the lab within two days of sampling for gas measurements using a Hewlett-Packard 5890 series II gas chromatograph (GC) that was fitted with a flame ionization detector (FID). Certified CO_2_ standards in 1.92 μl l-1 (China National Research Center for Certified Reference Materials, Beijing) were used for calibration. The fluxes of these samples were calculated using a linear regression of the gas concentration against time.

### Measurements of auxiliary factors

Air temperature (inside the chamber), soil temperature (10 cm depth, portable digital thermometer) and moisture (0–20 cm depth, Spectrum TDR300) were synchronously measured during each gas sampling event. Shoot biomass was measured by clipping canopy biomass at the ground level in six quadrats (1 m × 1 m) adjacent to the frames on the same day for each treatment (SG vs NG) at each site. The corresponding root biomass was also sampled using a stainless steel corer (7.0 cm in diameter). Plant biomass was oven dried at 70 °C to a constant weight.

### Calculations and statistical analyses

The gross ecosystem photosynthesis (GEP) was calculated using the formula: GEP = NEP + ER, and the canopy respiration (CR) was calculated using the formula: CR = ER − SR. The mean rate of all indices was the arithmetic average of all measurements in each growing season. All data are shown in mg CO_2_ m^−2^ h^−1^. For each sample, the data from both NG and SG plots were considered as paired data for each steppe type. For each growing season or all three years, the paired-sample t-test was used to determine the significance of each variable between no grazing and summer grazing for each steppe type. A multivariate general linear model was used to determine the relative contribution of steppe type, use pattern (no grazing and summer grazing), and year on the variation in NEP, GEP, CR and SR. Linear regression was performed to examine the single and combined effects of temperature and soil moisture (using the stepwise method) on seasonal rates of NEP, GEP, CR and SR at the steppe and regional levels (three steppes in total). Scatterplots with trend lines were used to display the effects of grazing on the seasonal relations between GEP and SR, and between GEP and CR at the steppe and regional levels. SPSS statistics 21 was employed to carry out the various statistical analyses, and Sigma Plot 10.0 was employed to draw the figures.

## Additional Information

**How to cite this article**: Hou, L. *et al*. Grazing effects on ecosystem CO_2_ fluxes differ among temperate steppe types in Eurasia. *Sci. Rep.* **6**, 29028; doi: 10.1038/srep29028 (2016).

## Figures and Tables

**Table 1:** Single or combined effects of air temperature (AT)/soil temperature (ST) and soil moisture (M) on the seasonal patterns of gross ecosystem photosynthesis (GEP), net ecosystem photosynthesis (NEP), canopy respiration (CR) and soil respiration (SR) under summer grazing (SG) and no grazing (NG) in the meadow steppe (MS), typical steppe (TS), and desert steppe (DS).

		GEP		CR		SR		
		AT	M	AT	M	ST	M	ST M
MS	NG	0.08	0.01	0.16	0.02	0.38**	0.27*	0.38(ST)**
MS	SG	0.20*	0.02	0.31**	0.00	0.52**	0.02	
TS	NG	0.19*	0.01	0.03	0.08	0.22*	0.11	
TS	SG	0.13	0.12	0.08	0.36**	0.19*	0.22*	0.41(ST M)**
DS	NG	0.00	0.05	0.01	0.05	0.00	0.30**	
DS	SG	0.04	0.10	0.00	0.19*	0.00	0.45**	

The data shown are the R^2^ of CO_2_ fluxes induced by the various factors; * and ** indicate significance levels of 0.05 and 0.01, respectively. No significant factors affected NEP; thus, the data are not shown.

**Table 2:** Effects of temperature [air temperature (AT), soil temperature (ST)], soil moisture (M), canopy biomass (CB) and root biomass (RB) on annual patterns of gross ecosystem photosynthesis (GEP), net ecosystem photosynthesis (NEP), canopy respiration (CR) and soil respiration (SR) under summer grazing (SG) and no grazing (NG), for the three steppe types pooled together.

		AT/ST	M	CB	RB
GEP	NG	−0.604**	0.045	0.203	0.034
	SG	−0.344*	0.104	0.419*	0.021
CR	NG	−0.128	0.581**	0.503**	0.020
	SG	0.006	0.743**	0.412*	0.153
SR	NG	0.411*	0.41*	0.686**	0.029
	SG	−0.009	0.646**	0.545**	0.022

The data are shown as the R^2^ of CO_2_ fluxes induced by the various factors, and “−” indicates negative relationships; * and ** indicate significance levels of 0.05 and 0.01, respectively.

**Table 3:** Contributions (R^2^) of steppe type (ST), use pattern (UP), and year (YR) to the spatiotemporal variances in the annual mean rates of net ecosystem photosynthesis (NEP), gross ecosystem photosynthesis (GEP), canopy respiration (CR) and soil respiration (SR).

	NEP(mg CO_2_m^−2^ h^−1^)			GEP(mg CO_2_m^−2^ h^−1^)			CR(mg CO_2_m^−2^ h^−1^)			SR(mg CO_2_m^−2^ h^−1^)		
	R^2^	C-R^2^	Sig.	R^2^	C-R^2^	Sig.	R^2^	C-R^2^	Sig.	R^2^	C-R^2^	Sig.
ST	0.373	0.373	0.002	0.658	0.658	0.001	0.437	0.437	0.001	0.725	0.725	<0.001
UP	0.002	0.375	0.722	0.005	0.663	0.658	0.044	0.482	0.135	0.006	0.730	0.576
YR	0.416	0.791	0.001	0.066	0.729	0.269	0.311	0.793	0.004	0.060	0.790	0.223

**Table 4:** **Soil properties (0**–**20** cm **layer) in the three steppe types.**

	Meadow steppe		Typical steppe		Desert steppe	
	NG	SG	NG	SG	NG	SG
Clay (%)	5.48 a		3.76 b		5.44 a	
Silt (%)	57.88 a		46.20 b		41.32 c	
Sandy (%)	36.64 b		50.04 a		53.24 a	
Bulk density (g cm^−3^)	0.99 b	1.12 ab	1.11 ab	1.09 ab	1.29 a	1.33 a
Total carbon (%)	2.26 a	2.33 a	1.63 b	1.46 b	1.22 b	1.12 b
Total nitrogen (%)	0.23 a	0.23 a	0.19 ab	0.18 ab	0.16 b	0.15 b
C/N	9.83 a	9.96 a	8.70 b	8.33 b	7.79 c	7.41 c
pH	7.85 ab	6.95 c	6.96 c	7.58 b	8.14 a	8.19 a
Ammonia (mg N.kg^−1^ soil)	2.69 b	3.11 a	2.42 bc	2.04 d	2.18 d	1.55 e
Nitrate (mg N.kg^−1^ soil)	8.73 bc	11.47 a	9.03 b	7.17 d	7.93 cd	5.23 e
AK (mg K. kg^−1^ soil)	413.14 a	224.27 b	282.11 a	250.84 a	238.84 a	175.61 b
AP (mg P. kg^−1^ soil)	6.33 a	3.43 a	3.56 a	3.22 a	5.72 a	4.51 a
MBC (mg C.kg^−1^ soil)	115.63 a	91.85 a	141.15 a	117.72 a	40.83 b	51.92 b
MBN (mg N.kg^−1^ soil)	13.10 b	10.68 b	17.34 a	12.47 b	1.73 c	3.99 c
Soil temperature 2012 (°C)	22.5b	23.7a	16.6d	16.5d	20.4c	21.4b
Soil temperature 2013 (°C)	20.0b	20.3b	15.8d	17.0c	22.0a	22.6a
Soil temperature 2014 (°C)	18.3b	18.9b	17.0c	17.5bc	23.8a	24.4a
Soil moisture 2012 (V/V%)	56.8a	46.3b	33.3c	31.5c	24.6d	25.4d
Soil moisture 2013 (V/V%)	47.8a	33.0b	19.2d	22.0c	19.8d	18.6d
Soil moisture 2014 (V/V%)	39.6a	29.5b	17.3c	19.3c	11.3d	12.3d

The data in a single row with different lower-case letters indicate significant differences (P < 0.05), based on Duncan’s multiple range tests. Abbreviations: C/N, total carbon/total nitrogen ratio; AP, available phosphorus; AK, available potassium; MBC, microbial biomass carbon; MBN, microbial biomass nitrogen.
